# Interpreting transcriptional changes using causal graphs: new methods and their practical utility on public networks

**DOI:** 10.1186/s12859-016-1181-8

**Published:** 2016-08-24

**Authors:** Carl Tony Fakhry, Parul Choudhary, Alex Gutteridge, Ben Sidders, Ping Chen, Daniel Ziemek, Kourosh Zarringhalam

**Affiliations:** 1 Department of Computer Science, University of Massachusetts Boston, 100 Morrissey Boulevard, Boston, 02125 USA; 2 Computational Sciences, Pfizer Worldwide Research & Development, Cambridge, USA; 3 Department of Engineering, University of Massachusetts Boston, Boston, 100 Morrissey Boulevard02125 USA; 4 Computational Sciences, Pfizer Worldwide Research & Development, Berlin, USA; 5 Department of Mathematics, University of Massachusetts Boston, 100 Morrissey Boulevard, Boston, 02125 USA

**Keywords:** Causal reasoning on biological networks, Inference on gene regulatory networks, Gene set enrichment analysis

## Abstract

**Background:**

Inference of active regulatory cascades under specific molecular and environmental perturbations is a recurring task in transcriptional data analysis. Commercial tools based on large, manually curated networks of causal relationships offering such functionality have been used in thousands of articles in the biomedical literature. The adoption and extension of such methods in the academic community has been hampered by the lack of freely available, efficient algorithms and an accompanying demonstration of their applicability using current public networks.

**Results:**

In this article, we propose a new statistical method that will infer likely upstream regulators based on observed patterns of up- and down-regulated transcripts. The method is suitable for use with public interaction networks with a mix of signed and unsigned causal edges. It subsumes and extends two previously published approaches and we provide a novel algorithmic method for efficient statistical inference. Notably, we demonstrate the feasibility of using the approach to generate biological insights given current public networks in the context of controlled in-vitro overexpression experiments, stem-cell differentiation data and animal disease models. We also provide an efficient implementation of our method in the R package QuaternaryProd available to download from Bioconductor.

**Conclusions:**

In this work, we have closed an important gap in utilizing causal networks to analyze differentially expressed genes. Our proposed Quaternary test statistic incorporates all available evidence on the potential relevance of an upstream regulator. The new approach broadens the use of these types of statistics for highly curated signed networks in which ambiguities arise but also enables the use of networks with unsigned edges. We design and implement a novel computational method that can efficiently estimate *p*-values for upstream regulators in current biological settings. We demonstrate the ready applicability of the implemented method to analyze differentially expressed genes using the publicly available networks.

## Background

The advent of cost-effective high-throughput functional genomics methods has spurred on the generation of transcriptional datasets in many diverse areas of biology. A common goal in the analysis of such data is to discover the regulatory pathways behind biomedical phenomena and as our understanding of these regulatory mechanisms increases, commercially and publicly available databases of regulatory interactions grow steadily. Ideally, a regulatory interaction implies a direction of causality, i.e. the perturbation of an upstream regulator causally leads to a downstream consequence. We consider two types of interactions in this work: (a) signed interactions that specify whether an increase in the upstream regulator causally leads to an *increase* or a *decrease* in the downstream entity, and (b) unsigned interactions that merely state that an upstream entity causally regulates a downstream entity, but do not specify the direction of effect. Throughout this paper, the word upstream is used to refer to regulators one step previous to a gene in a biological pathway. Commercial products, such as Qiagen’s IPA application (http://www.ingenuity.com/), are based on manually curated networks with a large number of signed causal relationships extracted from nearly 5 million findings [1]. At the time of writing, Qiagen’s webpage (www.ingenuity.com/ipa) lists more than 10,000 citations of biomedical articles making use of their commercial product on top of such a network. Unfortunately, such highly curated networks are not freely available to the academic community for further algorithmic development and generation of biomedical insights.

Several statistical approaches have been suggested to infer active upstream regulators from gene expression data based on a large set of *signed* causal interactions. The company Selventa Inc. pioneered the general approach [2]. Chindelevitch et al. [3] derived the exact null distribution for a plausible scoring scheme to rank putative upstream regulators. Kramer et al. [1] provide an approximation to this approach based on a normal distribution, which forms the basis for the popular IPA pathway analysis tool. Zarringhalam et al. [4] consider Bayesian approaches that also incorporate biological context into the inference procedure. Based on these algorithms, networks of biological interactions derived from commercial vendors such as Ingenuity (www.ingenuity.com) and Selventa (www.selventa.com) have been used to study processes as diverse as in vitro differentiation [5], modeling of cellular proliferation [6], and drug-induced liver injury [7].

In this work, we propose a new extended method to detect upstream regulators geared towards mixed networks paired with an efficient statistical inference approach. Importantly, we will demonstrate that the publicly available STRING database [8]^1^ has matured to a point to reproduce key findings from several previously published studies. The STRING10 Human database contains ∼200,000 molecular interactions of which ∼20*%* are either undirected (i.e. non-causal) or directed and not signed. In our method, we make use of both types of interactions.

The rest of the paper is structured as follows: we first present the intuition behind our new approach to analyze mixed networks and contrast it with previously proposed methods. In the following sections, we outline the ideas for efficient statistical inference and give a mathematical derivation to compute *p*-values based on the proposed statistics. Importantly, we demonstrate superior execution times even for the previously proposed approaches and show that the new statistic is preferable based on simulations. Finally, we demonstrate the biological plausibility of results based on publicly available networks in the context of controlled in-vitro overexpression experiments, stem-cell differentiation data and animal models of neuropathic pain. We close by summarizing our work and providing avenues for future extensions.

## Methods

### Approach

**Fig. 1:**
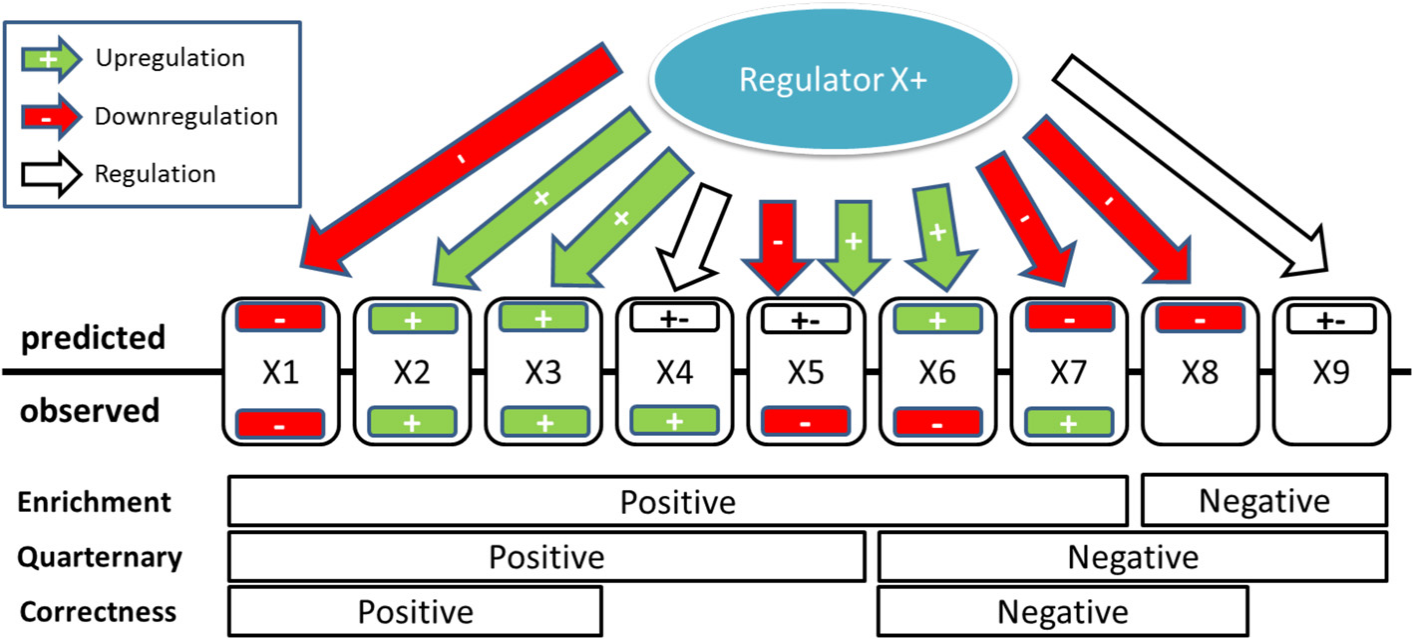
Schematic graph of an upstream causal regulator connected to a set of downstream transcripts. For each transcript, the *predicted* and *observed* direction of regulation are displayed. The bottom panel annotates positive and negative evidence for the activity of regulator *X* as defined for different scoring schemes discussed in the main text: **a** Enrichment score, **b** Quaternary score and **c** Correctness score

Our method will infer likely upstream regulators given (1) a set of up- and down-regulated transcripts from a specific biological experiment and (2) a mixed network of regulatory interactions potentially relevant in the current biological context. We define the network as a directed graph where the nodes are biological entities and the edges represent interactions between the entities. A causal signed edge in the network consists of a source node (typically proteins, compounds, miRNAs, etc) regulating the target node (typically transcripts). Signs + or − indicate up- or down-regulation respectively. An unsigned causal relation is an edge where the direction of regulation is either unknown or ambiguous. Ambiguity can arise when one source of information (e.g. a scientific article) describes an increasing regulatory relationship between two entities and another source postulates a decreasing one. This might be due to different biological contexts or simply erroneous findings in one of the articles. Figure 1 shows a schematic representation of a potential upstream regulator in a causal network with corresponding experimental data. Note that we assume a *positive* direction of regulation for the putative upstream regulator. All predictions flip if we have a negative direction of regulation.

In this work, we extend the ideas of [9] to include unsigned edges and derive the null distributions of the relevant test statistic for exact statistical inference. In addition, we provide a novel computational algorithm that runs significantly faster and has benefits even when using purely signed networks.

Given an upstream regulator *X* and the corresponding observed direction of regulation of the down stream nodes, we can construct a contingency table by tabulating all potential combinations of prediction and observation (cf. Table 1).

**Table 1:** Tabulation of predictions vs. observations for a given regulator and differentially regulated transcripts

	Observed +	Observed −	Observed 0	Total
Predicted +	*n*_++_	*n*_+−_	*n*_+0_	*q*_+_
Predicted −	*n*_−+_	*n*_−−_	*n*_−0_	*q*_−_
Predicted *r*	*n*_*r*+_	*n*_*r*−_	*n*_*r*0_	*q*_*r*_
Predicted 0	*n*_0+_	*n*_0−_	*n*_00_	*q*_0_
Total	*n*_+_	*n*_−_	*n*_0_	*N*

4×3 Contingency table

In Table 1, *q*_+_, *q*_−_, *q*_*r*_ and *q*_0_ denote the total number of +, −, unsigned (regulated) and 0 predictions respectively. Similarly, *n*_+_, *n*_−_, and *n*_0_ denote the total number of observed +, − and 0 perturbations according to the gene expression data. The entries of the table represent the agreement between the predictions made by the regulator and the actual observed values and correspond to several exemplary cases in Fig. 1. For instance, *X*1, *X*2 and *X*3 are all *correct* predictions in which the predicted and observed directions of regulation match exactly. These correspond to cells *n*_−−_ and *n*_++_ respectively. In contrast, *X*6 and *X*7 represent *incorrect* predictions and correspond to cells *n*_+−_ and *n*_−+_. *X*4 and *X*5 are cases in which the direction of regulation is unknown or ambiguous and the experimental data indeed show differential expression (cells *n*_*r*+_ and *n*_*r*−_). *X*8 and *X*9 are cases in which we would expect differentially expressed genes but don’t find any in our experiment. *X*8 corresponds to cell *n*_−0_ and *X*9 to cell *n*_*r*0_. Differentially expressed transcripts that are not predicted to be regulated by the upstream regulator under consideration are captured in cells *n*_0+_, *n*_0−_, and *n*_00_ (zero predictions correspond to nodes with no edge to the regulator).

Note that the total number of predicted and observed altered genes are determined a priori according to the gene expression data and the causal graph. This implies that the margins of the table for each upstream regulator are fixed and the table is completely determined by the upper left 3×2 corner. The probability of the table *T* under the null model that the predictions made by the regulator (or equivalently the observed gene expression values) are distributed at random given the constraints on the margins of the table, can be computed by a generalization of the hypergeometric probability mass function. Essentially the probability is obtained by the ratio of the total number of permutations (i.e., randomizations) of the gene expression values that do not change the table, and the total number of possible permutations while keeping the margins fixed, i.e., *P*(*T*) is given by

1 $$ \frac{\binom{q_{+}}{n_{++},n_{+-},n_{+0}} \binom{q_{-}}{n_{-+},n_{--},n_{-0}} \binom{q_{r}}{n_{r+},n_{r-},n_{r0}}\binom{q_{0}}{n_{0+},n_{0-},n_{00}}}{\binom{N}{n_{+},n_{-},n_{0}}}  $$

Here *N* denotes the sum of the row (equivalently column) margins. The terms in the numerator of the above fraction are the multinomial coefficients and represent the total number of identical tables under random permutations of gene values. We refer to this number as *D-value* and denote it by *D*[+,−,*r*]. The denominator is the total number of possible permutations (i.e., total number of tables with the same margins) and is denoted by *D*_*tot*_.

Next, we show how to assign various scoring schemes to the table and compute their statistical significance. Under the null hypothesis and for any given test statistic *S*(*T*), the significance of an observed value *S*_0_ of the test statistic is computed by summing the probability of the tables with the same or a more extreme values of the test statistic, i.e., $\sum _{S(T) \geq S_{0}} P(T)$.

The scoring schemes are defined based on the available information on the direction of regulation in the causal graph. Table 1 shows the most general scenario of mixed networks and subsumes important special cases. For example, if sign information is ignored or not available, the first two rows in table 1 will be equal to zero. Any differentially expressed transcript that is predicted to be regulated by *X* is *positive evidence* for an active regulator *X*. Consequently, the score for the goodness-of-fit of the predictions to the observed experimental data is given by the *enrichment* score (cf. Enrichment in Fig. 1).

2 $$\begin{array}{*{20}l} ES(T) &= n_{r+} + n_{r-}. \end{array} $$

Under the null hypothesis, this test statistic has the following probability mass function.

3 $$\begin{array}{*{20}l} P(ES = S_{0}) &= \sum\limits_{ES(T) = S_{0}} \frac{\binom{q_{r}}{n_{r+},n_{r-},n_{r0}}\binom{q_{0}}{n_{0+},n_{0-},n_{00}}}{\binom{N}{n_{+},n_{-},n_{0}}} \end{array} $$

Using Vandermonde’s identity, it is not difficult to show that the above probability mass function is equivalent to

4 $$\begin{array}{*{20}l} P(ES = S_{0}) &= \frac{\binom{q_{r}}{n_{r+} + n_{r-}}\binom{q_{0}}{n_{0+}+n_{0-}}}{\binom{N}{n_{+} + n_{-}}}. \end{array} $$

This amounts to Fisher’s exact test, a statistic that is routinely used for gene set enrichment tests [10]. The test was also proposed by [2] to analyze causal signed networks. However, this score does *not* use any information on direction of regulation and is unable to predict the likely direction of regulation of an upstream regulator. Nevertheless, in networks of unsigned edges it may be the only available option.

In contrast, Chindelevitch et al. [9] propose the *correctness* statistic that focuses on signed edges only. It scores an upstream regulator and its putative direction of regulation by considering the difference between *correct* and *incorrect* predictions. As the approach considers three different states for predictions. i.e. correct, incorrect, and not regulated, it was also called the *ternary* score. Transcripts are considered positive evidence for the upregulation of regulator *X* if their predicted direction of regulation matches their actual direction of differential expression. Similarly, they count as *negative evidence* if the directions do not match (cf. Correctness in Fig. 1). As this method ignores all unsigned edges, the third row of the table is assumed to be zero. The goodness-of-fit score of the table in this case is

5 $$\begin{array}{*{20}l} CS(T) &= n_{++} + n_{--} - (n_{+-} + n_{-+}). \end{array} $$

The significance of the above statistic (*correctness score*) can be computed in a similar fashion as for the enrichment score, i.e. $\sum _{CS(T) = s_{0}} P(T)$.

Finally, we can introduce our new score which is applicable in mixed networks, but retains the ability to assess directionality for regulators if sign information is available. As this score considers not only correct, incorrect and not regulated transcripts as the *ternary* or *correctness* score does, but also ambiguously regulated transcript, we name it the *quaternary* score. In this case, we combine the evidence metric of the enrichment score for the unsigned interactions with the metric of the correctness score for the signed interactions. Starting from an enrichment perspective, the score adds information on likely directionality by penalizing transcripts with incorrectly predicted direction of differential expression. From a correctness score perspective, we include information on activity of the regulator by counting evidence from unsigned interactions (cf. Quaternary in Fig. 1). Hence, this score can be viewed as an intermediate, matching the enrichment score when no information on the direction of regulation is available and transitioning to the correctness score when all the interactions are signed. In most publicly available networks, it is expected that there will be a mixture of the both types of interactions (signed and unsigned). This score is defined to be

6 $$\begin{array}{*{20}l} QS(T) &= n_{++} + n_{--} + n_{r+} + n_{r-} - (n_{-+} + n_{+-}). \end{array} $$

The significance of the quaternary score can be computed in a similar fashion to the other scores. The degrees of freedoms in the randomization of the tables are 1, 4 and 6 in the enrichment, correctness and quaternary cases, respectively. This results in time complexity of *O*(*n*), *O*(*n*^4^) and *O*(*n*^6^) for computing the entire score distributions. In particular the *O*(*n*^4^) and *O*(*n*^6^) complexities are impractical for most applications. It is important to note that in all cases above, the scores contain all entries of the table corresponding to the degrees of freedom. Including additional terms in the score will not change the distribution but merely shifts it.

In [3], the authors presented an algorithm for approximating the significance of the correctness score. Essentially, their algorithm approximates the sum by identifying classes of tables with low probabilities and discarding them from the computations. Due to the nature of their algorithm, the entire distribution of the scores must be approximated before the significance of the observed score can be computed. Since we are primarily interested in computing the *p*-values, approximation of the entire distribution is unnecessary and we only need to approximate the probability of the scores that are as or more extreme than the observed score. In the next section we show how we can exploit the structure of the probability distribution of the scores to achieve a more efficient algorithms for enrichment, correctness, and quaternary scores alike.

### Model

In this section, we outline the theoretical foundation of our algorithm. The probability of scores of tables with given fixed margins follows a specific pattern that can be exploited to approximate the *p*-value of an observed score *s*_0_ in an efficient manner. For the ease of presentation, we describe the method in the 3×3 setting (i.e., Correctness statistic). The method is naturally generalized to the 4×3 case (Quaternary statistic). If the margins of the table are fixed, the table will be completely determined by 4 cells in the table, i.e., there are a total of 4 degrees of freedom. For example, the table can be parametrized by the upper left 2×2 corner of the table, i.e, *n*_++_, *n*_−−_, *n*_−+_, *n*_+−_. We may replace one of these parameters (for example *n*_+−_) with the score of the table. For a set of fixed margins, we can enumerate all tables in a specific order. For instance we can impose the following dictionary ordering on the entries of the table:

7 $$ n_{++},\,n_{--},\,n_{-+},\,n_{+-},\,n_{+0},\,n_{-0},\,n_{0+},\,n_{0-},\,n_{00}  $$

**Fig. 2:**
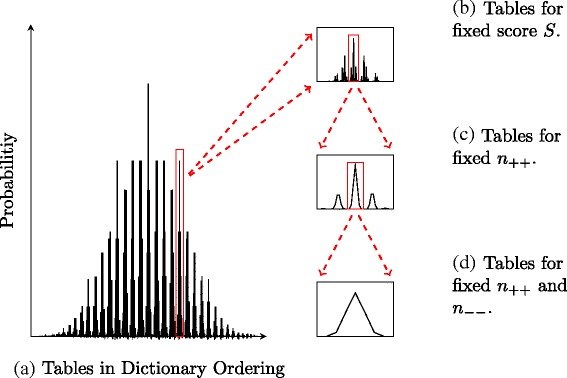
**a** Probabilities of matrices in the dictionary ordering *S,n*_++_, *n*−−, *n*_−+_, *n*_+−_, *n*_+0_, *n*_−0_, *n*_0+_, *n*_0−_, *n*_00_ along the x-axis where all margins equal are equal to 15. Figure **b**, **c** and **d** show the graph at increasingly higher resolutions

We emphasize again that the tables are determined once 4 parameters are known. Figure 2 shows probabilities of tables ordered by *S*, *n*_++_, *n*_−−_ and *n*_−+_ on the x-axis. As can be seen, there is a repeated pattern of probabilities for classes of tables defined by these parameters. For example, in the class of tables with a fixed score *S*, *n*_++_ and *n*_−−_, we see that the distribution is unimodal (See Fig. 2d). Indeed, we will show later that any set of tables with fixed *S*, *n*_++_ and *n*_−−_ are at most bimodal with the two modes being directly next to each other when ordered in the dictionary ordering of (7).

We now give an informal description of the algorithm that will help in motivating the theoretical arguments. Let *M*[*S*], *M*[*S,n*_++_] and *M*[*S,n*_++_,*n*_−−_] denote the categories of tables with fixed parameter values as indicated by the argument. More precisely, *M*[*S*] is the set of tables for a given fixed *S*, *M*[*S,n*_++_] is the set of tables for a given fixed *S* and *n*_++_, and *M*[*S,n*_++_,*n*_−−_] is the set of tables for a given fixed *S*, *n*_++_ and *n*_−−_. There are several tables in each of these categories of various probabilities, ranging from a unimodal graph in *M*[*S,n*_++_,*n*_−−_] class to a graph consisting of several peaks in *M*[*S,n*_++_] and *M*[*S*] classes. Note that for a fixed *S*, the class *M*[*S*] contains the tables in *M*[*S,n*_++_] and *M*[*S,n*_++_,*n*_−−_] classes (See Fig. 2–b–d). Our algorithm essentially identifies the peaks of the *M*[*S*] class for each possible value of *S*. By adding the probabilities of tables in a local neighborhood of each peak in the *M*[*S*] class, the probability of *S* is approximated, from which the *p*-value can be approximated within any user-defined tolerance. In order to achieve this, all the peaks in *M*[*S*] (which contains the subclasses *M*[*S,n*_++_] and *M*[*S,n*_++_,*n*_−−_]) as well as the tables in a local neighborhood around the peaks need to be identified. We achieve this task through a series of adjustments to the entries of tables that can efficiently transition between the tables in each category. The starting point of the algorithm is the table with the minimum possible score *S*_*min*_. There exists only one such table and hence must also be the table of maximum probability within the *M*[*S*_*min*_] class. The algorithm proceeds to find the table with the maximum probability in the next class (i.e, next possible score) by adjusting the entries of the current maximum probability table in a way that a) moves the table to the next desired class and b) the adjustments perturb the table as minimally as possible. The intuition behind this is that the tables with maximum probability in each class correspond roughly to the tables where the entries are most evenly distributed given the margin and class constraints. The reason for this is that the probability of a table is maximized when the numerator in Eq. (1) is maximized which happens approximately when the entries of the table are as close to each other as possible. Hence starting from *M*[*S*_*min*_], we need to adjust the table minimally to move to the table with maximum probability in the next class *M*[*S*_*next*_]. Once the table with maximum probability in *M*[*S*_*next*_] is identified, we identify other peaks in subclasses *M*[*S*_*next*_,*n*_++_] and *M*[*S*_*next*_,*n*_++_,*n*_−−_] through similar adjustments (Moves between and within the different classes will be discussed in detail later in the section - see Definition 3).

The process of adjusting the tables can be viewed as a permutation process where the symbols +, − and 0 are re-distributed into three buckets of sizes *q*_+_, *q*_−_ and *q*_0_ (right margin of the table, corresponding to the +, − and 0 predictions made by the regulator). The total number of symbols are given by *n*_+_, *n*_−_ and *n*_0_ respectively (bottom margin of the table, corresponding to +, − and 0 genes under the regulation of the regulator). Each such distribution is essentially moving the symbols from one bucket to another which results in a table of the same margins but with possibly different entries. We refer to such permutations as *Moves*. The simplest moves are those which interchange 2 different elements from two different buckets. For example, we can remove a + from the *q*_+_ bucket and place it into the *q*_−_ bucket; remove a − from the *q*_−_ bucket and place it into *q*_+_ bucket. Note that we may need to combine several such moves to obtain a table within the desired class. Also, note that the table of the maximum probability in the *M*[*S*] class automatically defines *M*[*S,n*_++_] and *M*[*S,n*_++_,*n*_−−_] classes, i.e., classes in which the table resides. Once the algorithm is at this table, all the tables within *M*[*S,n*_++_,*n*_−−_] class with probability higher than a pre-specified threshold are enumerated via valid moves and their probabilities are added to the probability of the score. As we will see later, there is only one move that generates all tables in *M*[*S,n*_++_,*n*_−−_] class. For thresholding we use the maximum *D*-value of all tables (independent of the parameters) times some *ε* (e.g., 1e-16), i.e., any table in the *M*[*S,n*_++_,*n*_−−_] class with probability below this threshold value is discarded. This is the same thresholding scheme which was proposed in [3]. Next, the algorithm moves to the table of maximum probability in *M*[*S,n*_++_,*n*_−−_+1] as well as *M*[*S,n*_++_,*n*_−−_−1] classes and the same process is repeated until all the *n*_−−_ values are exhausted, at which point the algorithm moves to the next *M*[*S,n*_++_+1] and *M*[*S,n*_++_−1] classes and repeats the process once more. Once the *M*[*S,n*_++_] is exhausted the algorithm moves to the next score class toward the tail of the distribution to which the observed score is closer. At each stage of the algorithm, if the table of maximum probability in the *M*[*S*] or *M*[*S,n*_++_] classes has probability below the threshold, the entire classes are discarded, which results in significant speed up of the algorithm. If no thresholding is applied the algorithm will be of complexity *O*(*kn*^3^) where *k* is the number of considered scores as opposed to the *O*(*n*^4^) complexity of the brute force algorithm. However, in practice the approximation scheme will result in complexity much lower than *O*(*kn*^3^). We now formalize the definition of moves and prove a few results that are essential for the description of the algorithm.

#### **Definition 1**

A transposition is a move in which an element *x* is moved from a bucket *q*_*i*_ to a bucket *q*_*j*_, *i*≠*j*, and an element *y* is moved from the bucket *q*_*j*_ to the bucket *q*_*i*_. We denote this transposition by (*q*_*i*_,*x,q*_*j*_)(*q*_*j*_,*y,q*_*i*_).

Transpositions are essentially the minimal permutations of the symbols {+,−,0} in the buckets *q*_+_, *q*_−_ and *q*_0_ that result in tables with the same margins. Note that transpositions change a given table only if *x*≠*y*. An equivalent way of describing the transpositions is as follows. Each transposition corresponds to a 3×3 matrix as follows. Starting from a zero 3×3 matrix whose columns correspond to symbols +, − and 0 and whose rows correspond to buckets *q*_+_, *q*_−_ and *q*_0_, we place a −1 entry where the element is being removed from the corresponding bucket and a +1 where the element is being added to the other bucket. Other elements of the matrix remain 0. For example *τ*=(*q*_+_,+,*q*_−_)(*q*_−_,0,*q*_+_) corresponds to the matrix $M =\left [\begin {array}{ccc} -1 & 0 & 1 \\ 1 & 0 & -1 \\ 0 & 0 & 0 \end {array} \right ]$. Note that applying the transposition *τ* to a given 3×3 table *T* is equivalent to adding the matrix representation of *τ* to *T*, i.e., *τ*(*T*)=*M*+*T*. Moreover, for appropriately chosen positive integers *k*, the operation *kM*+*T* (i.e *τ*^*k*^(*T*)) will result in a table with equal margins as in *T*. Here, appropriate means that the entries of the resulting table must remain non-negative. The operation *kM*+*T* is a permutation (move) that may not necessarily correspond to a transposition. There are a total of 18 possible transpositions, each corresponding to a transposition matrix *M*_1_,*M*_2_,...,*M*_18_. It can be shown that for any tables *T* and *T*^′^ with the equal margins, there exist a linear combination of the transposition matrices such that

8 $$  T' = \sum\limits^{18}_{i=1} k_{i}M_{i} + T,  $$

where *k*_*i*_≥0 [11]. In particular, this implies that any arbitrary permutation (move) *σ* can be written as a linear combination of transposition matrices i.e $\sigma = \sum ^{18}_{i=1} k_{i}M_{i}$. In other words, *σ* can be decomposed as a product of transpositions which keeps the matrix margins fixed and all the entries of the table non-negative. Moreover, since matrix addition is commutative, the order in which the transpositions are applied is irrelevant. This is not to say that two moves are commutative as elements of the permutation group, but for a given move, the overall order in which one applies the transpositions to a table is of no importance and the resulting table will always be the same. Next, we need to define the notion of evenness in the distribution of the entries of a given table with fixed margins. Evenness is used as a proxy for the table of maximum probability in each category. Define the auxiliary function *d*(*x,y*,*z*)=(*x*−*y*)^2^+(*x*−*z*)^2^+(*y*−*z*)^2^. Minimizing this function will aid in obtaining the most evenly divided table. For example, if we were to distribute the +, − and 0 symbols in the *q*_+_ bucket as evenly as possible, we would need to minimize *d*(*n*_++_,*n*_+−_,*n*_+0_) i.e the number of +, − and 0 has to be as close to each other as possible. Similar reasoning holds for distributing the symbols to other buckets. In general, the measure of evenness can be computed as follows. Let *T* be a 3×3 table and let

$$\begin{array}{*{20}l} d(T) &= d(n_{++},n_{+-},n_{+0}) + d(n_{-+},n_{--},n_{-0}) \\ & \quad + d(n_{0+},n_{0-},n_{00}) + d(n_{++}, n_{-+}, n_{0+}) \\ & \quad + d(n_{+-}, n_{--}, n_{0-}) + d(n_{+0}, n_{-0}, n_{00}). \end{array} $$

Then the most evenly divided table of given fixed margins is the one with minimum *d* value.

Let *M*^′^ be the most evenly divided table of given fixed margins and let *τ* be a transposition and *σ* be an arbitrary move that includes *τ* as a factor in its decomposition. If *σ* is not a transposition different from *τ* then we have *d*(*M*^′^)≤*d*(*τ*(*M*^′^))≤*d*(*σ*(*M*^′^)). The first part of the inequality follows from the fact that *M*^′^ is the most evenly divided table, hence any move applied to the table will deviate it from evenness. The second part of the inequality holds since the decomposition of *σ* is not a transposition different from *τ* and (as a product of transpositions) *σ* contains *τ* therefore adjustments applied by *σ* are at least as large as adjustments applied by *τ*.

As stated before, the algorithm proceeds from the table with minimum score and identifies tables of maximum probability in subsequent categories. In order to make such transitions, we need to define the notion of principal moves that transition from the table at the current stage of the algorithm to the most probable table in the next desired category. First, we need the definition of a minimal move.

#### **Definition 2**

Minimal moves are the moves generated by considering all possible combinations of transpositions without replacement.

For example, consider the move *τ*_1_=(−1,0,0,1,0,0,1,−1,0) applied according to the dictionary ordering (7), i.e., *τ*_1_ adjust the entries by the indicated amounts in the same dictionary ordering. In matrix form $\tau _{1} =\left [ \begin {array}{ccc}-1 & 1 & 0 \\ 0 & 0 & 0 \\ 1 & -1 & 0 \end {array}\right ]$. Moreover, the move *τ*_1_ can be decomposed into a product of transpositions as *τ*_1_=(*q*_0_,−,*q*_+_)(*q*_+_,+,*q*_0_). Since each transposition is repeated once, *τ*_1_ is a minimal move. On the other hand, consider *τ*_2_=(−1,0,−2,1,0,2,3,−1,−2). In matrix form, $\tau _{2} =\left [ \begin {array}{ccc}-1 & 1 & 0 \\ -2 & 0 & 2 \\ 3 & -1 & -2 \end {array}\right ]$ and can be decomposed as *τ*_2_=(*q*_−_,+,*q*_0_)^2^(*q*_0_,0,*q*_−_)^2^(*q*_0_,−,*q*_+_)(*q*_+_,+,*q*_0_)=(*q*_−_,+,*q*_0_)^2^(*q*_0_,0,*q*_−_)^2^*τ*_1_. Note that *τ*_2_ is not a minimal move since some of the transpositions are repeated more than once.

Both of these moves keep the margins of the table fixed as can be readily seen from their matrix representations. Since *τ*_2_ is a product of more transpositions (specifically transpositions that don’t cancel each other out) than *τ*_1_, then *τ*_1_ will adjust the table less. It should be noted that any arbitrary move *σ* can always be decomposed as *σ*=*σ*_*m*_*σ*_*n*_ where *σ*_*m*_ is a minimal move and *σ*_*n*_ is a product of transpositions that is not necessarily minimal. This is because $\sigma = \prod _{l \in I} \tau _{l}$, is a product of transpositions and since the order of applying the transpositions does not matter, we can rearrange the transpositions to attain *σ*=*σ*_*m*_*σ*_*n*_. From this, it follows that the minimal moves are precisely those where *σ*=*σ*_*m*_*σ*_*n*_ with *σ*_*n*_=1. Here 1 represents the identity transposition, corresponding to the zero 3×3 matrix.

**Fig. 3:**
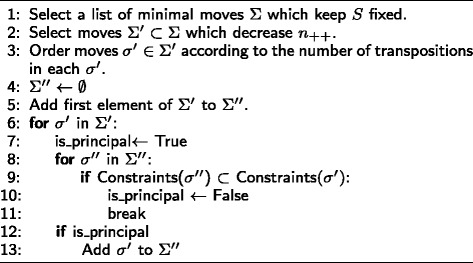
Algorithm for computing principal moves which decrease *n*_++_ and fix *S*

The algorithm relies on the fact that we can move to the table of highest probability in each class (In fact, it is possible to move between any two tables *T* and *T*^′^ through a series of moves (8)). For example, if we want to move from the table of highest probability in *M*[*S,n*_++_], to the table of highest probability in the next class in the dictionary ordering, we can generate a set of moves to make this jump directly. We refer to these moves as principal moves. If there are multiple moves that can achieve this task, we select one at random. The principal moves for decreasing *n*_++_ are generated using the algorithm in Fig. 3. Note that the algorithm in Fig. 3 uses the function Constraints (*σ*) which returns the set of indices at which the move *σ* is negative, where indices range from 1 to 9 in the dictionary ordering (7).

**Fig. 4:**
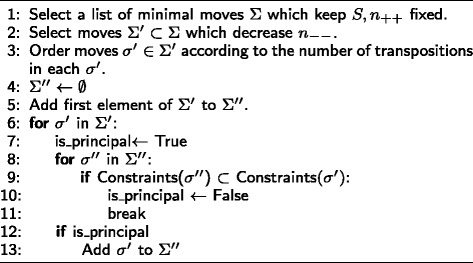
Algorithm for computing principal moves which decrease *n*_−−_ and fix *S,n*_++_

Let *M*_*max*_[*S,n*_++_] be the table of maximum probability in *M*[*S,n*_++_] and let *M*_*max*_[*S,n*_++_−*l*] (*l*≥1) be a table of maximum probability of class *M*[*S,n*_++_−*l*] where *n*_++_−*l* is the next possible value of *n*_++_ in the dictionary ordering of (7). The algorithm in Fig. 3 generates the list of moves *σ*^″^∈*Σ*^″^ s.t for some *σ*^″^ we have *σ*^″^(*M*_*max*_[*S,n*_++_])=*M*_*max*_[*S,n*_++_−*l*]. That is to say if there is indeed a valid matrix *M*_*max*_[*S,n*_++_−*l*] then there must be some *σ*^″^ that can take us to *M*_*max*_[*S,n*_++_−*l*]. We note again that minimal moves contain the set of all possible combinations of constraints that can arise in any arbitrary move. For instance, in the previous example, if *τ*_2_ is applicable then *τ*_1_ is also applicable since Constraints (*τ*_1_)⊂ Constraints (*τ*_2_). We should also note that the principal moves change *n*_−−_ by at most 1, therefore *l*=1. However, we did use the notation *l* to stress the fact that for some degrees of freedom, the next value in the dictionary ordering may be greater than 1 (e.g *n*_*r*+_ in the 4×3 case). We can also generate the principal moves which increase *n*_++_ with a slight modification to the algorithm in Fig. 3. In a similar fashion, the principal moves which find the next possible value of the next degree of freedom *n*_−−_ can be calculated. The algorithm in Fig. 4 will generate the moves which decrease *n*_−−_ and keep *S* and *n*_++_ fixed.

**Fig. 5:**
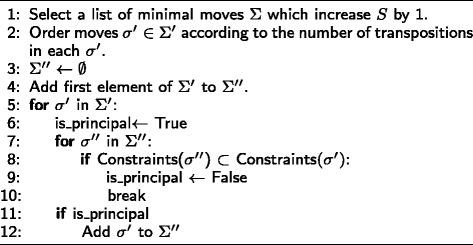
Algorithm for computing principal moves which increase *S* by 1

The only principal move in *M*[*S,n*_++_,*n*_−−_] is *σ*_1_=(0,0,−1,1,−1,1,1,−1,0). This move decreases *n*_−+_ and keeps *S*, *n*_++_ and *n*_−−_ fixed. Similarly $\sigma ^{-1}_{1} = (0, 0, 1, -1, 1, -1, -1, 1, 0)$ reverses the effect of *σ*_1_. Moreover, it can be shown that the only principal *S* increasing moves that exist, increase the score by 1, 2 and 4. Hence the scores in the domain of the Correctness statistic are differenced by 1, 2 and 4. The algorithm in Fig. 5 is a slight modification of Algorithms 1 and 2 that generates the principal moves which increase the score by 1.

Principal moves which increase the score by 2 and 4 are generated in a similar way. Next we present a few facts about tables with maximum and minimum possible scores that we need in our algorithm. These will be the tables on the tails of the distribution. In order to get the table with maximum score, we have to put the maximum number of + symbols in the *q*_+_ bucket, maximum number of − symbols in the *q*_−_ bucket and the remaining + and − symbols in the *q*_0_ bucket. The rest of the entries are determined by the margins of the table. Therefore, we have to set the entries as *n*_++_= min{*q*_+_,*n*_+_}, *n*_−−_= min{*q*_−_,*n*_−_}, *n*_0+_= min{*q*_0_,*n*_+_−*n*_++_} and *n*_0−_= min{*q*_0_−*n*_0+_,*n*_−_−*n*_−−_}. In particular this shows that there is only one table with maximum score. Similarly there is only one table with minimum score and the entries of the table are given by *n*_+−_= min{*q*_+_,*n*_−_}, *n*_−+_= min{*q*_−_,*n*_+_}, *n*_0+_= min{*q*_0_,*n*_+_−*n*_−+_} and *n*_0−_= min{*q*_0_−*n*_0+_,*n*_−_−*n*_+−_} and there is only one table with minimum score.

#### **Theorem 1**

In the *M*[*S,n*_++_,*n*_−−_] class, there exists at most two matrices with maximum probability.

#### *Proof*

We know that there exists at least one table *T*∈*M*[*S,n*_++_,*n*_−−_] with highest probability. Let *T*^′^∈*M*[*S,n*_++_,*n*_−−_] be such that $T' = \sigma ^{-1}_{1}(T)$. Consider the ratio of the probabilities of *T*^′^ and *T*:

9 $$\begin{array}{*{20}l} & \frac{D[n_{++},n_{--},n_{-+}+1,n_{+-}-1]}{D[n_{++},n_{--},n_{-+},n_{+-}]}  \\ &= \frac{n_{+-}n_{-0}n_{0+}}{(n_{-+} + 1)(n_{+0} + 1)(n_{0-} + 1)}  \end{array} $$

10 $$\begin{array}{*{20}l} & = \frac{(n_{++} + n_{--} - S - n_{-+})(q_{-} - n_{--} - n_{-+})}{(n_{-+} + 1)(q_{+} - 2n_{++} - n_{--} + S + n_{-+} + 1)}  \\ & \times \frac{(n_{+} - n_{-+} - n_{++})}{(n_{-} - 2n_{--} - n_{++} + S + n_{-+} + 1)}  \end{array} $$

We see from Eq. (10) that as we increase *n*_−+_ the probability becomes smaller than or equal to *Prob*(*T*). Moreover, since *n*_−+_+*n*_+−_=(*n*_−+_+1)+(*n*_+−_−1), *n*_+0_+*n*_−0_=(*n*_+0_+1)+(*n*_−0_−1) and *n*_0+_+*n*_0−_=(*n*_0+_−1)+(*n*_0−_+1), we see that Eq. (9) can equal 1 only if *n*_−+_=*n*_+−_−1, *n*_+−_=*n*_−+_+1, *n*_+0_=*n*_−0_−1, *n*_−0_=*n*_+0_+1, *n*_0+_=*n*_0−_+1, *n*_0−_=*n*_0+_+1. Hence we see that there can be at most two tables with maximum probability and the proof is complete. □

Similarly, it is not difficult to see that for *M*[*S,n*_++_,*n*_−−_] there exist at most two tables which are most evenly devided. We can now state the algorithm which computes the probability of a score formally.

**Fig. 6:**
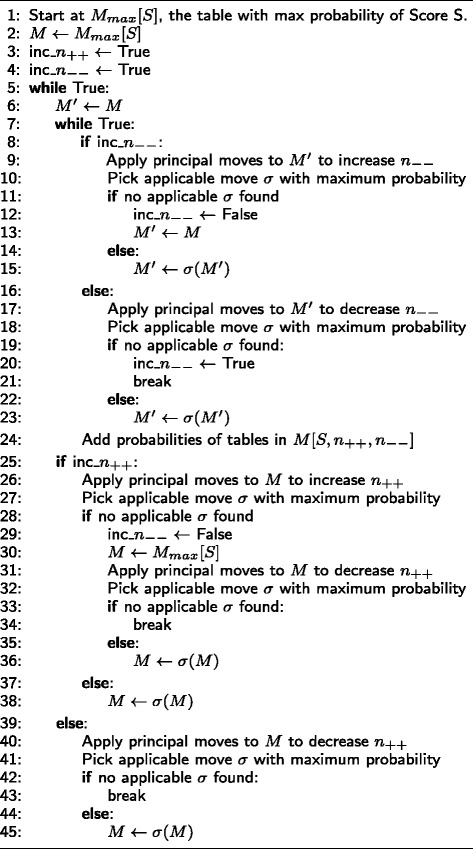
Algorithm for computing the probability of a score S

The algorithm in Fig. 6 shows that for computing the probability of a score *S*, we have to iterate through all the possible values of *n*_++_ and *n*_−−_. We apply the principle moves to find out if it is possible to increase or decrease the values of *n*_++_ and *n*_−−_. The starting values of *n*_++_ and *n*_−−_ are those of the matrix with highest probability *M*_*max*_[*S*] for a given score *S*. To get *M*_*max*_[*S*], we have to start at the matrix of minimum score, then apply principal moves to get the matrix with highest probability of the next score. The procedure is repeated until we reach our target score. When increasing or decreasing the values of *n*_−−_, we choose the principal move *σ* which maximizes the probability and leaves *n*_++_ fixed. This method can naturally be generalized to the 4×3 case. The only difference is that the 4×3 case can have more than one table of minimum score. When this happens, the tables of minimum score have 1 degree of freedom, so the table of highest probability can be found by computing the mode of the hypergeometric distribution. In practice, the algorithm in Fig. 6 as implemented in the R package is modified to allow discarding matrices below a certain threshold, and thus approximate the probability or *p*-value of a score similar to [3]. When increasing/decreasing the values of *n*_++_ and *n*_−−_, we can discard the classes *M*[*S,n*_++_] and *M*[*S,n*_++_,*n*_−−_] which have a maximum probability less than a certain threshold. Similarly, since the probabilities in *M*[*S,n*_++_,*n*_−−_] are at most bimodal with the two modes being directly next to each other, we can stop increasing/decreasing *n*_−+_ when the probability falls below a certain threshold. Thresholding significantly speeds up the algorithm since there are many tables of negligible probabilities.

### Data processing

All gene expression data were normalized and differentially expressed genes were computed using the R limma package. Unless otherwise stated, in all analyses we used a minimum 1.3 fold change and <0.05 FDR corrected *p*-value to detect differential expression. Differentially expressed genes were assigned to +1 (up-regulated) or −1 (down-regulated) according to the sign of the fold change.

## Results and discussion

All results are based on the R package QuaternaryProd which implements the above outlined strategy to compute Enrichment, Correctness, and Quaternary *p*-values given differentially expressed genes and a mixed input network. The package is written in Rcpp [12] (C++ for R) and is available to download from Bioconductor. We will first demonstrate the quality and speed of the approximation approach. We will then show that the Quarternary statistic compares favorably with previous statistics in a simulation setting. Finally, we demonstrate the ability of our algorithm to recover plausible biological hypothesis using the publicly available STRING10 [8] network in the context of controlled in-vitro overexpression experiments, stem-cell differentiation data and animal models of neuropathic pain.

### Benchmarking and quality of approximation

We benchmarked our algorithm against previous implementation of the Correctness score [3]. To assess the speed of the algorithms, we generated 1000 tables with values ranging from 0 to 200 for the *q*_+_, *q*_−_, *n*_+_, *n*_−_ margins and values ranging from 1000 to 5000 for *q*_0_. The *q*_*r*_ is set to 0 in Correctness score calculations. The range of the values were selected to reflect typical gene expression and network connectivity values. For each table the *p*-values of the score and the elapsed time were calculated for both algorithms. The threshold value was set to 1e-16 in both algorithms. On average our algorithm runs 45× faster than that of [3] with a maximum speed up to a 1000× depending on where the observed score falls in the distribution. Additionally we tested the speed of the algorithm in computing the significance of the Enrichment score as compared with the Fisher’s exact test implemented in the R fisher.test function. The time taken in both algorithms are very comparable and typically ≤0.05 sec.

Next we assessed the speed and accuracy of the algorithm in computing the significance of the Quaternary score. Both speed and the accuracy depends on the selected value of the threshold. For example, setting the threshold value to 0 will result in a brute force computation of the *p*-value (maximum accuracy), but slow runtime (*O*(*n*^6^) complexity). On the other extreme, setting the threshold to 1 will run the fastest, but with very low accuracy.

### Results on simulated data

In order to illustrate the performance of the three scoring statistics (QS, CS, ES) in networks with various degrees of ambiguity, we consider a hypothetical network consisting of 20,000 transcripts and 5,000 potential upstream regulators. We assume an *active upregulated* regulator *R*1 with 100 downstream transcripts. We also consider an *inactive* regulator *R*2 which shares the same set of 100 downstream transcripts. *R*1 and *R*2 differ in the direction of regulation for 50 of their 100 downstream transcripts, i.e. they share a certain degree of their downstream response, but also differ substantially. All other regulators will not be considered here and will, in general, overlap only to a small degree with R1’s and R2’s downstream transcripts. We reflect their presence by choosing a multiple testing corrected significance threshold of 0.05/5000=10^−5^. We then simulate 1000 expression data sets based on R1’s active state by randomly assigning expression changes to 15 % of R1’s down stream transcripts correctly and 5 % incorrectly. In addition, we randomly add 200 downregulated transcripts and 300 upregulated transcripts that reflect other ongoing changes in the system, potentially related to other regulators in the network.

**Fig. 7:**
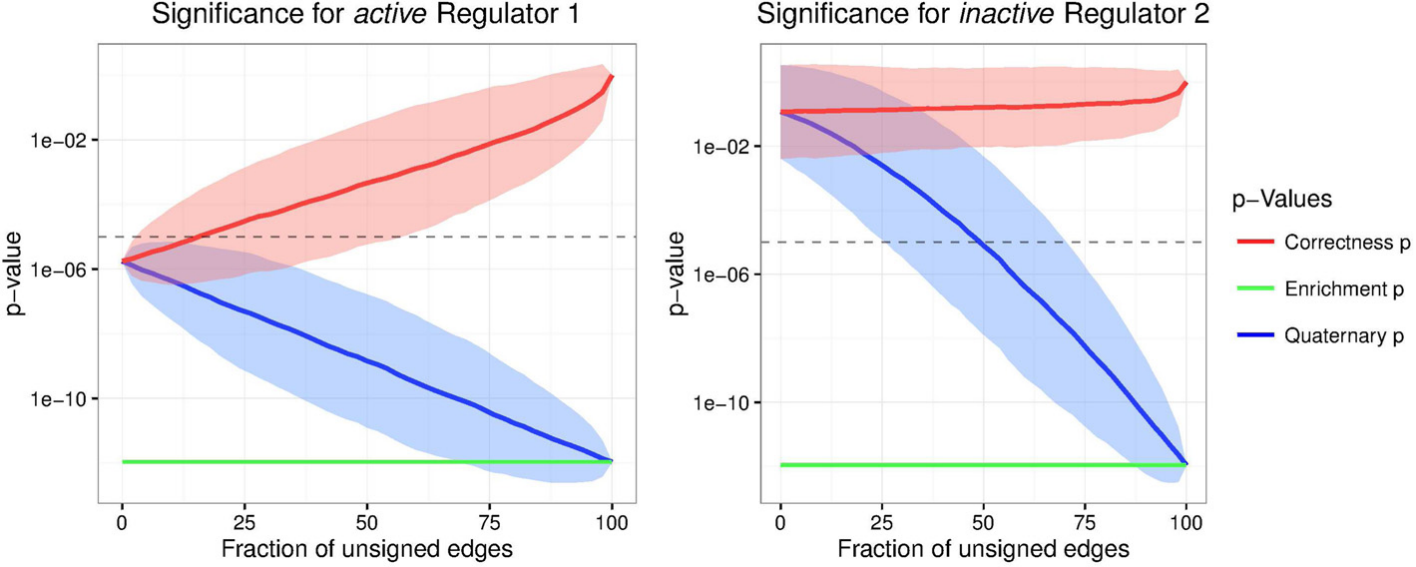
This figure shows simulations results in recovering upstream regulators using the quaternary (*blue*), correctness (*red*) and ES (*green*) scoring statistics. Thin lines represent individual simulations and thick lines represent the averages across the 1000 simulations. The x-axis represents the percentage of ambiguous edges in the network and the y-axis represents the *p*-value on a log scale. Figure on the left depicts an active regulator R1 simulated to be up-regulated. Figure on the right depicts an inactive regulator R2 that shares downstream transcripts with R1 but has a different direction of regulation for 50 *%* of its edges. The dotted black line marks a significance level of 1e-5. Further details in the main text

Each expression data set is generated based on the *true* underlying network structure. To reflect our incomplete knowledge of direction of regulation we subsequently set a larger and larger fraction of edges to an unsigned state and compute *p*-values for all three scoring statistics. Note that we do not consider deletion or insertion of random edges here. Such analysis has been conducted in [3]. Our focus is on the presence of ambiguous edges in the network. Simulation results are depicted in Fig. 7. Thin lines represent individual simulations and thick lines represent the averages across the 1000 simulations.

Firstly, note that the quaternary *p*-value reduces to the correctness *p*-value in the case of no ambiguity and to the enrichment *p*-value for complete ambiguity. Furthermore, the enrichment *p*-value is always constant as only the direction of regulation changes in our simulation. In our example, the enrichment *p*-value would always correctly flag R1 and incorrectly flag R2 as active. The correctness *p*-value correctly identifies R1 as active and R2 as inactive when full information on direction of edges is available. However, the performance deteriorates quickly when more and more ambiguous edges are present and no regulators are detected as active. Our new quaternary statistic is able to optimally make use of the available information. It is able to predict the correct activity status for the regulators even with significant ambiguity. Specifically, it retains the ability to detect R2’s inactive state even with little information of directionality of regulation and will, therefore, lead to more precise hypotheses for follow-up, if the direction of regulation information in the network is trustworthy. In contrast, if our knowledge of direction of regulation is faulty, enrichment scores might give superior results in some cases. Similarly, if unsigned edges are not trustworthy, the correctness score would be preferable to the quaternary score. In general, we assume a network topology as well as specified direction of regulation to reflect a (potentially noisy) version of the underlying true network. In that setting, the quaternary score should be the statistic of choice. Detailed characteristics of the simulation depend on the chosen patterns values, but the outlined patterns remain valid for a wide range of parameter choices.

### Recovering known stimuli in an in-vitro setting

To demonstrate the performance of our method in conjunction with the publicly available STRING10 network, we use the same validation set as suggested by [3]. This dataset was derived from [13] in which they used recombinant adenoviruses to infect non-cancerous human mammary epithelial cells with a construct to overexpress specific oncogenes. This provides an excellent test dataset as there are clear single perturbations to recover. As in [3], we focus on the c-Myc, H-Ras, and E2F3 expression signatures. Differential gene expression analysis of these data sets resulted in 118, 202, and 241 differentially expressed genes respectively. Table 2 shows the top 5 regulators predicted by the algorithm along with the FDR corrected *p*-values of the scoring schemes. Note that the *p*-values differ from the original publication due to the applied multiple testing correction and the use of a different network. In the c-Myc experiment, the algorithm recovers the up-regulation of Max as the top hypothesis. It has been demonstrated that oncogenic activity of c-Myc requires dimerization with Max [14]. Myc is the second top hypothesis. In this case the CS *p*-value is more significant than the QS *p*-value. There are a total number of 318 genes downstream of c-Myc in the network. Of these 105 are ambiguous, only one of which is connected to a differentially expressed gene. For the E2F3 experiment, E2F1 is returned as the top hypothesis. E2F1 and E2F3 are close family members and have a very similar role as transcription factors that function to control the cell cycle and are similarly implicated in cancer [15]. Note that in contrast to QS, the CS algorithm is unable to recover this hypothesis at a significant FDR corrected *p*-value. The fraction of unsigned edges implicating E2F1 is relatively high at 58 % and this result demonstrates the advantages of the QS algorithm in such cases. In the H-Ras experiment, EGR1 is the top hypothesis returned by the algorithm with a very significant quaternary *p*-value. EGR1 is a key regulator of oncogenic processes and is downstream of, and positively regulated by, HRAS [16], fitting the direction of regulation observed in our results. In summary, we are able to recover either the known perturbation, a paralogous gene, or a downstream mediator of the perturbed gene’s activity. In all cases the biology behind the expression signature is sufficiently explained, and we would expect the accuracy of our predictions to improve as coverage of the interaction network expands.

**Table 2:** Top 5 regulators predicted by the algorithm in over expression experiments [13]

		c-Myc						H-Ras						E2F3		
Name	Regulation	QS	CS	ES		Name	Regulation	QS	CS	ES		Name	Regulation	QS	CS	ES
MAX	up	2e-3	1e-2	1e-3		EGR1	up	7e-6	2e-2	8e-5		E2F1	up	3e-6	2e-1	4e-5
MYC	up	8e-2	4e-3	6e-1		JUN	up	1e-3	9e-4	9e-5		ADORA2B	up	3e-2	1e-1	1e-2
DNAJC3	down	1e-1	4e-1	4e-1		GAST	up	2e-3	2e-4	2e-3		RBX1	down	3e-2	2e-1	2e-2
E2F2	up	1e-1	6e-1	3e-1		CXCR2	up	6e-3	6e-3	1e-2		CDKN1A	down	7e-2	9e-1	1e-4
E2F3	up	1e-1	7e-1	3e-1		CSF2	up	7e-3	8e-3	1e-2		SKP2	down	1e-1	2e-1	1e-1

FDR corrected *p*-values of the 3 scoring schemes are listed: Quaternary score (QS), Correctness score (CS) and Enrichment score (ES)

### Factors for stem cell directed differentiation

Directed differentiation of stem cells to specific cell types is an important challenge in regenerative medicine. Using a time course of stem cell differentiation to a pancreatic endocrine fate we previously showed that the CS statistic was able to identify Interleukin 6 (IL6) as a novel secreted factor involved in this process [5]. However this result was only obtained with the CS statistic in conjunction with a proprietary network. Repeating the analysis with the STRING10 network we are only able to obtain significant results (FDR < 0.01) with the QS statistic. Table 3 shows the top 5 regulators.

**Table 3:** Top regulators predicted by the algorithm. FDR corrected *p*-values of the 3 scoring schemes are listed

Name	Regulation	QS	CS	ES
AURKB	Up	9.4e-4	2.2e-2	4.2e-3
GAST	Up	2.4e-3	2.2e-2	2.1e-4
IL6	Up	6.8e-3	7.5e-2	6.1e-3
FGF2	Up	8.1e-3	5.7e-2	1.1e-4
NEUROG3	Up	8.9e-3	4.1e-2	1.3e-2

Pancreatic endocrine maturation

Aurora Kinase B (AURKB), Gastrin (GAST), IL6, FGF2 and NEUROG3 are all predicted to be up-regulated during endocrine specification. Of these IL6, NEUROG3 and Gastrin have known roles in pancreatic endocrine formation. We consider this good evidence that the QS statistic provides significant additional power to identify upstream regulators of stem cell differentiation compared to CS and that this allows the method to be successfully used in conjunction with freely available causal networks. Next we turned to a model of early forebrain and eye field development (Surmacz et al., 2012). Neural progenitor cells were replated from a fibronectin matrix to CellStart and treated with the secreted factor Activin A for 4 days in order to generate retinal precursors. Microarrays were used to profile the transcriptome of the cells before and after treatment [ArrayExpress: E-MTAB-4259]. There were a total of 1730 differentially expressed genes which were used as input to the QS statistic in conjunction with the STRING10 network. The top 5 most significant hypotheses are shown in Table 4.

**Table 4:** Top regulators predicted by the algorithm. FDR corrected *p*-values of the 3 scoring schemes are listed

Name	Regulation	QS	CS	ES
VEGFA	Up	2.4e-5	2.3e-5	9.0e-7
PTK2	Up	2.4e-5	7.7e-5	2.5e-4
TGFB1	Up	2.4e-5	2.4e-4	4.3e-6
BMP4	Up	3.0e-5	1.3e-2	1.2e-5
ATF2	Up	4.7e-5	1.4e-4	2.4e-4

Early forebrain and eye field development

Of these TGFB1 (transforming growth factor beta) is the primary ligand of the canonical transforming growth factor beta signaling pathway that is also activated by Activin A [17]. We consider therefore that while the method is unable to recover the precise treatment applied to the cells it has successfully identified the correct activated pathway. The activation of PTK2 (also known as focal adhesion kinase) is also expected and consistent with the replating of the cells onto a new extracellular matrix as PTK2 is directly downstream of signals initiated by cell-ECM interactions [18]. The activation of VEGFA (vascular endothelial growth factor A) and BMP4 (bone morphogenic protein 4) signaling is unexpected as neither of these factors are present in the exogenously provided media after replating. Returning to the original transcription data revealed that both genes encoding these factors were expressed by the cells at least 2 fold higher post-treatment (FDR < 0.0001) suggesting that these pathways are activated endogenously within the culture in response to the replating and Activin A treatment. BMP4 in particular is known to play a key role in eye development consistent with the overall hypothesis that the cells are being driven to an ocular fate [19]. ATF2 activation is also novel in this system. There is no concomitant change in expression of the ATF2 gene as we observe for VEGFA and BMP4, but there is evidence in other models that activation of ATF2 via phosphorylation by p38 kinase can occur in response to Activin A treatment [20], suggesting that this transcription factor may play an important role in mediating the downstream effects of Activin A.

### An animal model of neuropathic pain

Characterisation of animal disease models is an important class of biomedical experiment and we wished to test whether our method could provide insight into regulatory pathways using data from such a model. Neuropathic pain is a significant chronic pain state caused by injury or other damage, e.g. inflammatory, to the nervous system. 20 % of the European population is thought to suffer from chronic pain, with 5 % exhibiting chronic neuropathic pain [21]. We previously reported a gene expression signature from a model of neuropathic pain [22] [ArrayExpress: E-MTAB-2260] and here we apply causal reasoning to identify the underlying molecular basis for the establishment of a chronic neuropathic pain state.

Of the top hypotheses, the majority are immunological (See Table 5). The most significant causal hypothesis is IL1B, a key cytokine involved in the development of neuropathic pain and which has been shown to directly enhance excitatory currents within neurons of the DRG [23, 24]. The third causal hypothesis, IL6, has also been shown to directly modulate neuronal activity, reducing inhibitory currents [25]. Both hypotheses fit with the known underlying pathology of neuropathic pain whereby a large pro-inflammatory response occurs in response to injury, leading to long term maladaptive plasticity that maintains a chronic neuropathic pain state [26].

**Table 5:** Top regulators predicted by the algorithm. FDR corrected *p*-values of the 3 scoring schemes are listed

Name	Regulation	QS	CS	ES
IL1B	Up	7.0e-5	1.9e-2	9.9e-6
JUN	Up	4.2e-4	4.1e-3	7.5e-4
IL6	Up	4.5e-4	1.9e-2	3.0e-4
FOSL1	Up	2.0e-3	1.7e-2	3.9e-3
FGF2	Up	3.6e-3	1.8e-2	6.8e-3

Animal models of neuropathic pain

## Conclusions

In this work, we have closed an important gap in utilizing causal networks to analyze differentially expressed genes. Our proposed Quaternary test statistic incorporates all available evidence on the potential relevance of an upstream regulator as exemplified in Fig. 1 and can be seen as a generalization of the well-known Enrichment score used in gene set enrichment approaches [10] and the Correctness statistic suggested in [3]. This new approach broadens the use of these types of statistics for highly curated signed networks in which ambiguities arise but also enables the use of networks with unsigned edges, i.e. mixed networks, which are prevalent in the academic sector. A direct estimation of the null distribution of the proposed statistic would lead to a prohibitively slow *O*(*n*^6^) algorithm. In this work, we design and implement a novel computational method that can efficiently estimate *p*-values for commonly occurring tables in current biological settings. Most importantly, we demonstrate the ready applicability of the implemented method to analyze differentially expressed genes using the publicly available STRING10 network. While the precision of inference is not as high as with commercially available networks at this point, the derived putative upstream regulators describe relevant biology and can readily be used for follow-up hypothesis testing. We see future work for the inference of upstream regulators given mixed networks primarily in the area of plausible and efficient incorporation of biological context and the construction of higher level models. While Zarringhalam et al. [4] provided an initial proposal for Bayesian inference incorporating context on signed networks and Kramer et al. [1] extend upstream regulator discovery beyond the first layer, many questions around efficient inference, publicly available data and best practices remain to be solved.

With this work we hope to broaden the appeal of prior causal network methods in the academic community by demonstrating that biologically plausible inference is possible with currently available networks and the R package QuaternaryProd we provide with this paper. We believe this will generate biologically testable hypotheses in specific use cases, but also spur method development to tackle outstanding questions in this field.

## Endnote

^1^ In this paper, we consider STRING10 as available under a Creative Commons Attribution 3.0 License.

## Abbreviations

QS: Quaternary score
CS: Correctness score
ES: Enrichment score
FDR: False discovery rates
